# The Involvement of Hemocyte Prophenoloxidase in the Shell-Hardening Process of the Blue Crab, *Callinectes sapidus*

**DOI:** 10.1371/journal.pone.0136916

**Published:** 2015-09-22

**Authors:** Javier V. Alvarez, J. Sook Chung

**Affiliations:** Institute of Marine and Environmental Technology, University of Maryland Center for Environmental Science, 701 E. Pratt Street, Columbus Center, Baltimore, Maryland, United States of America; Uppsala University, SWEDEN

## Abstract

Cuticular structures of arthropods undergo dramatic molt-related changes from being soft to becoming hard. The shell-hardening process of decapod crustaceans includes sclerotization and mineralization. Hemocyte PPO plays a central role in melanization and sclerotization particularly in wound healing in crustaceans. However, little is known about its role in the crustacean initial shell-hardening process. The earlier findings of the aggregation of heavily granulated hemocytes beneath the hypodermis during ecdysis imply that the hemocytes may be involved in the shell-hardening process. In order to determine if hemocytes and hemocyte PPO have a role in the shell-hardening of crustaceans, a knockdown study using specific *CasPPO-hemo-dsRNA* was carried out with juvenile blue crabs, *Callinectes sapidus*. Multiple injections of *CasPPO-hemo-dsRNA* reduce specifically the levels of *CasPPO-hemo* expression by 57% and PO activity by 54% in hemocyte lysate at the postmolt, while they have no effect on the total hemocyte numbers. Immunocytochemistry and flow cytometry analysis using a specific antiserum generated against CasPPO show granulocytes, semigranulocytes and hyaline cells as the cellular sources for PPO at the postmolt. Interestingly, the type of hemocytes, as the cellular sources of PPO, varies by molt stage. The granulocytes always contain PPO throughout the molt cycle. However, semigranulocytes and hyaline cells become CasPPO immune-positive only at early premolt and postmolt, indicating that PPO expression in these cells may be involved in the shell-hardening process of *C*. *sapidus*.

## Introduction

Crustaceans, like other ecdysozoans, experience repeated molting processes during their life cycle, as they complete their growth, sexual maturation, and reproduction [1,2]. Successful molting is achieved through the coordinated actions of multiple hormones and neurohormones [3–7]. The cuticle undergoes dramatic structural and morpohological changes during the molt cycle, from being soft to becoming hard.

The hardening process of the crustacean new cuticle differs from that of insects and involves two sequential processes: sclerotization and mineralization [8–12]. The sclerotization occurs in the new soft and pliable cuticle immediately after ecdysis [13–15]. Mineralization, as the second step, incorporates calcium carbonate into the protein matrices of the cuticle in 2.5 hrs after ecdysis [8,9,14,16–21].

In insects, sclerotization involves both prophenoloxidases (PPOs) and laccases that cross-link chitinous materials to protein fibers in the matrices of the cuticle through the oxidation of phenol substrates [10,11,22–24]. In decapod crustaceans, there is no report on the presence of laccase as yet, whereas the PPO present in the hemocytes is primarily known for immunity responses [25–30]. Earlier studies, however, imply that the hemocytes may be involved in the shell-hardening process in crustaceans [31–33]. The aggregation of heavily granulated hemocytes are found beneath the hypodermis during ecdysis [31,32]. These findings imply that crustacean PPO may be transported into the new cuticle for sclerotization [31,32], similar to the ones reported in insects [34,35].

The hemolymph of decapod crustaceans carryies at least three types of hemocytes [36–38]: granulocytes; semigranulocytes; and hyaline cells. The hemocytes, as a cellular source of PPO, vary by species and molt stage. The granulocytes and semigranulocytes are the exclusive PPO sources at the intermolt of *Homarus americanus*, *Panulirus interruptus*, *Loxorhynchus grandis*, *Pacifastacus leniusculus*, *Penaeus japonicus*, *Procambarus clarkii*, and *Scyonia ingentis* [36,37,39–41]. The hyaline cells are the source of PPO during the ecdysis of *Uca pugilator* [31]. In *P*. *interruptus* and *L*. *grandis*, some hyaline cells are also present with PO activity, along with granulocytes [36]. These reports indicate that PPO may be produced by a specific cell type(s) at different molt stages.

Interestingly, the total hemocyte numbers and hemocyte PO activity change during the molt cycle of *Callinectes sapidus* [33]. When considered that cellular sources of PPO vary by molt stage [31,36,37,39–41], the changes in the PO activity during the molt cycle of *C*. *sapidus* may be derived from different types of hemocytes. More specifically, if the PPO expressed in hemocytes is involved in the initial shell-hardening process, we hypothesized that there may be differences in the population structure of hemocytes at postmolt, compared to other molt stages.

Herein, we report that during the molt cycle, there are changes in the types of hemocytes that are responsible for PPO expression. To further define the role of hemocyte PPO in the shell-hardening process, a knockdown experiment, specifically using a multiple administration of *CasPPO-dsRNA* injections, has been carried out. The effects of *dsRNA* injections are determined on the levels of *CasPPO-hemo* transcripts with a qPCR assay and of PPO protein in hemocytes using immunocytochemistry (ICC) and flow-cytometry. More importantly, the cuticle hardness of these animals has been measured at postmolt.

## Materials and Methods

### Animals

Juvenile *C*. *sapidus* crabs (15–30 mm carapace width, CW) were obtained from the blue crab hatchery [Aquaculture Research Center, Institute of Marine and Environmental Technology (IMET), Baltimore, MD, USA]. The animals were reared in individual compartments in recirculated, aerated artificial seawater (25 ppt; 22°C) as described [42–44]. Juveniles with ~80–90 mm CW were molt-staged by following the criteria as described prior to experiments [45]. All animals (both males and females) at intermolt stages were used, unless stated otherwise.

### Identification of hemocyte types in the hemolymph of *C*. *sapidus* during the molt cycle

#### Cytology

First, in order to identify hemocyte types, the hemolymph of the animals (n = 3 at intermolt; n = 3 at premolt) at different molt stages were withdrawn into a 1 ml syringe (23 G needle) containing a fixative (4% PFA in 10 mM cacodylate buffer) as described [33] at 1:1 ratio (v:v). Hemolymph smears were prepared as described [37] and stained with hematoxylin (1 min) and eosin (4 sec). The 6 slides (intermolt and premolt) were examined immediately after staining and digitally photographed under a compound microscope (National Microscopes). The images of hemocytes were measured for their diameter (mean ± SE μm) using AmScope MT software (AmScope MT) with an assumption that cells are round. The hemocyte types were identified by following the criteria as stated [37,38] and the properties of these cells were detailed as listed in Table 1.

**Table 1:** Properties of the different hemocyte types of *C*. *sapidus* and other decapod crustaceans.

Hemocyte type	Characteristics under a light microscope	Reference (species)
Granulocytes	Contain many large and a few small granules. Spindle, round or oval form	Wenli and Shield, 2007 (*C*. *sapidus*)
	Large eosinophilic granules. Low nuclear/cytoplasmic ratio. Mainly spherical shapes. The granules present in various degree of density that are compacted or fused into a homogeneous matrix	Lanz et al., 1993 (*P*. *clarkii*)
Semigranulocytes	Contain many small and a few large granules. Shapes and sizes vary a spindle, oval or round shape from 12 to 20 μm	Wenli and Shield, 2007 (*C*. *sapidus*)
	Presence of small, round or slightly oval, eosinophilic granules. Ovoid and fusiform cells. Low nuclear/cytoplasmic ratio. Round or spindle shape, but most often oval	Lanz et al., 1993 (*P*. *clarkii*)
Hyaline cells	Contain a few or no granules in the cytoplasm. Cells have a spindle or round form. Cell sizes with 9–18 μm	Wenli and Shield, 2007 (*C*. *sapidus*)
	Pleomorphic: flat pseudopodia normally extending from the hemocyte surface. Cytoplasm without granules and high to low nuclear/cytoplasmic ratio. Nucleus with well-defined heterochromatin	Lanz et al., 1993 (*P*. *clarkii*)

#### Flow cytometry

The types of hemocytes present in juvenile animals at different molt stages (n = 6–18) were also identified using flow cytometry. The hemocytes fixed as stated above were stained with SYBR-Green I (2X) nucleic acid staining (FMC BioProducts) as described [46] at 1:100 ratio (v:v = SYBR-Green I:sample). After 10 min incubation at RT, 50 μl of each sample was analyzed using a C6 CFlow flow cytometer (Accuri Cytometers), with setting at a media fluidic rate (35 μl/min) and a core size of 16 μm at FL1-H and SSC-H. The types of hemocytes were characterized by their sizes (FSC-H) and cytosolic complexities and granularities (SSC-H).

### Production of a rabbit-anti-CasPPO-hemo serum (α-CasPPO-hemo)

In order to generate the antiserum specific to CasPPO-hemo, the following two regions: V_421_ESRGQKNQL_430_ and V_199_SRDRKGELF_208_ that are located in between the hemocyanin domains were selected from the putative CasPPO sequence (GenBank accession no. AGE48302.1). These two regions are also on the surface of PPO molecule (Phyre^2^; http://www.sbg.bio.ic.ac.uk/phyre2/). A cysteine residue was added at the C-terminus of these peptides: V_421_ESRGQKNQL_430_C and V_199_SRDRKGELF_208_C and synthesized (Peptide 2.0 Inc).

Two different conjugation methods were carried out similarly as described [47]: m-maleimidobenzoyl-N-hydroxysuccinimide ester (MBS) conjugating the peptides to bovine thyroglobulin (bTG, Sigma) at a molar ratio of 50:1:15 (peptide: bTG: MBS) and 1-Ethyl-3-[3-dimethylaminopropyl]-carbodiimide hydrochloride (EDC) at a molar ratio of 50:1:560 (peptide:bTG: EDC) [48]. At the end of conjugation reactions (overnight at 4°C), 90% of each conjugated material was mixed and lyophilized for a rabbit antiserum production (Proteintech Group). The remaining 10% was dried in a SpeedVac (Jouan) and used for pre-absorption controls. The rabbit antiserum (α-CasPPO-hemo) was further characterized for a dilution factor, specificity and cross-reactivity using western blot analysis of hemocytes lysate supernatant (HLS) (S1 and S2 Figs and S1 Protocol).

### Effect of Cas*PPO-hemo-dsRNA on* the initial shell-hardness

#### CasPPO-hemo-dsRNA production

The template for *dsRNA* (243 bp) that excluded hemocyanin domains of CasPPO-hemo was generated by amplification of hemocyte cDNA with double-strand (ds) *CasPPO-hemo* primers (Table 2) and purified using Qiagen Gel extraction kit. The template DNAs were *in vitro* transcribed using a TranscriptAid™ T7 High Yield Transcription kit (Fermentas) as described [49]. *CasPPO-hemo*-*dsRNA* were diluted in 0.2 μm filtered crustacean saline solution containing phenol red at 0.001% [50] to give a final concentration at 0.1 μg/μl. Phenol red was added to monitor the delivery of injection materials into the animals [50].

**Table 2:** List of primers used for *dsRNA* template amplification and qPCR assay.

Primers	5’ to 3’ nucleotide sequence
*CasPPO*3F6-QF	CACCTCTTCATCCATCACAAACTC
*CasPPO*5R6-QR	CAACCACACCCACAGAAGTTAAAG
*CasPPO-ds3F9*	TAATACGACTCACTATAGGGCTTCGTCTACAGCATCAAGGTG
*CasPPO-ds5R9*	TAATACGACTCACTATAGGGTCCAAGTCACGGAAGGTAAAGT
*CasBurs-ds3F*b	TAATACGACTCACTATAGGTACTAGAACGTACGGTGTTGAATGCGC
*CasBurs-ds5R*b	TAATACGACTCACTATAGGTACTTTACCGGGTCGAGTCGCCACACTTG
*CasBurs-ds3F*a	TAATACGACTCACTATAGGTACTGACGAGTGTTCCCTGCGGCCTGT
*CasBurs-ds5R*a	TAATACGACTCACTATAGGTACTCTCAGAAAGGGAACGCTGTCCATTG

Q = qPCR primers; ds = *dsRNA* primer with T7 promotor sequence italicized.

#### CasPPO-dsRNA injection and levels of CasPPO-hemo transcripts

The crabs at intermolt stage (85.5 ± 2.3 mm CW, n = 7) were injected with 10 μg of *CasPPO-hemo-dsRNA* every other day until ecdysis (a total of 20–28 injections). Two control groups (88.0 ± 2.1 mm CW, n = 14) were injected with 100 μl of crustacean saline (control saline, n = 7) and 10 μg of *bursicon* α+β *dsRNA* (*burs* α+β = control *dsRNA*, n = 7). Bursicon that involves in shell-hardening process of insects [51], is also found in *C*. *sapidus* [42]. The templates for *CasBurs-*α and-β *dsRNAs* were generated by amplification of thoracic ganglia complex cDNA (α = 363 bp; β = 348 bp) using specific *dsRNA* primers listed in Table 2. Purification of templates and *in vitro* transcription were carried out as described above.

In order to determine the levels of *PPO* and PO activity at postmolt, the hemolymph samples were collected after crabs completed the expansion of the body at 24 hrs after ecdysis [15]. The hemolymph samples (400 μl) were collected for RNA extraction [33] and hemocyte lysate supernatant (HLS) as described [33]. The expression levels of *CasPPO-hemo* were estimated using the qPCR assay same as described [33] using the primers as listed in Table 2.

#### Levels of CasHLS-PO activity and CasPPO-hemo protein

The PO activity of CasHLS was determined by measuring the formation of dopachrome from L-3,4 dihydrophenylalanine (L-dopa) using a modified assay as described [33]. Briefly, HLS samples (25 mg HLS in 50 μl working buffer = 10mM sodium cacodylate, 5mM calcium chloride, pH 7.1) were preincubated with 50 μl of trypsin solution (1 mg/ml working buffer) for 15 min at 37°C. Subsequently, 100 μl L-dopa (3 mg/ml working buffer) were added and further incubated for 20 min at 37°C. The absorbance was measured at 490 nm (Molecular Devices). The data are shown as mean ± SE (n = 6–7) of PO activity (OD/mg HLS protein).

For the presence of CasPPO-hemo protein in hemocytes, 100 μl of hemolymph of the experimental animals at 24 hrs after ecdysis were collected as described above. The amounts of CasPPO-hemo in hemocytes were analyzed by the fluorescent intensity of the positive signal by α-CasPPO-hemo through an ICC analysis (described below) using ImageJ [52]. The all ICC images of hemocytes were taken using the same microscopic settings. The hemocytes were also analyzed for their cytosolic complexities using a C6 CFlow flow-cytometer (Accuri Cytometers). Additionally, CasPPO-hemo was detected in CasHLS using a western blot analysis (S1 Protocol).

#### Hardness of postmolt cuticle of *C*. *sapidus*

After ecdysis, the cuticle hardness of each crab was measured using a hand-held Shore-type durometer (00 gauge, Instron) as described [16,42] during the first 48 hrs after ecdysis, the period which includes early postmolt stages as described [15]. The hardness of the carapace was measured on mesogastric area (S3 Fig). The rates of hardness vary depending on the part of cuticle [16]. Therefore, the hardness was measured at 6–8 different spots of the cuticle of each animal and the average of these values was used. The data are shown as mean ± SE (n = 5–7) of durometer units (DU) as described [16].

### Differential and total hemocyte counting during the molt cycle

The different types of hemocytes and total hemocytes (DHC and THC) were counted using flow cytometry. The hemocytes from juvenile blue crabs at different molt stages (n = 6–18) were stained with SYBR-Green I (2X). The types of hemocytes were distinguished by their sizes (FSC-H) and cytosolic complexities (SSC-H). The data are presented as mean ± SE (number of cells/ml hemolymph).

### Identification of CasPPO-hemo in hemocytes during the molt cycle

#### Immunocytochemistry (ICC)

Hemolymph (50 μl) that was sampled and fixed from the animals at the following molt stages: intermolt, early premolt, late premolt, and postmolt (each, n = 5–6) as described above, was centrifuged for hemocyte collection [33]. The pelleted hemocytes were resuspended in 50 μl of a fixative solution (4% PFA in cacodylate buffer 10mM, pH 7.1). A drop of the samples (~5–8 μl) was smeared on a slide glass and air-dried for 20–30 min at RT [53]. These slides were immersed in 100% methanol for 30s, rinsed in milliQ water for 2 min and incubated for 10 min in PTX1 solution (PBS containing 0.25% Triton X-100 and 5% dimethyl sulfoxide, DMSO [53,54]). After rinsing the slides in milliQ water three times (5 min each), they were blocked with blocking buffer (PTX 1 containing 2% BSA) overnight at 4°C. Then, the slides were incubated with α-CasPPO-hemo (1:200 dilutions in the blocking buffer) for 30 min at RT and were washed 5 times in PBS (5 min each). The incubation with Alexa Flour goat anti-rabbit IgG (Life Technologies) at 1:100 dilution was carried out for 30 min at RT under darkness. After washing 5 times in PBS, the slides were briefly post-fixed in the fixative solution as above and washed with PBS. Finally, the slides were mounted with a ProLong Gold antifade reagent with DAPI staining (Life Technology). The hemocytes were examined for their subtypes under a fluorescent microscope (Axioplan, Zeiss) and the images were captured with a digital camera (AxioCam MRc, Zeiss). Staining intensity were further analyzed and calculated using Zen 2012 and ImageJ [52].

For the pre-absorption control, synthetic CasPPO peptides (10 nmol each) were incubated with α-CasPPO-hemo (a final dilution at 1:10) overnight at 4°C through gentle mixing on a magnetic stirrer. It was then diluted to a final dilution of 1:200, the same as the α-CasPPO-hemo prior to hemocyte incubation.

#### Flow cytometry analysis

The hemocytes collected from the crabs at intermolt (n = 8) and postmolt (n = 6) stages were fixed and washed in 1 ml PTX2 (0.1M phosphate buffer containing 0.5% Triton-X and 2% BSA and DMSO is removed from the buffer solution improving cell suspension in the samples for flow cytometry) and incubated for 15 min at RT for blocking. Then, the hemocytes were centrifuged (800 g for 10 min, 4°C) and incubated in α-CasPPO-hemo for 30 min at RT [55]. The samples were washed twice in 1 ml Dulbecco’s Phosphate Buffer Saline (PBSG) as described [55] by gentle pipetting, and shaking on a rotational shaker for 5 min. The incubation with the secondary antibody was carried out as above and the samples were washed once in 1 ml PBSG, and finally resuspended in 100 μl PBSG. Forty μl of each sample were analyzed in a C6 Flow Cytometer (Accuri Cytometers) under the conditions described as above.

In each flow cytometric analysis, fixed hemocytes were washed once and resuspended in 100 μl PBSG as negative controls. The positive staining of hemocytes with α-CasPPO-hemo was established and confirmed with negative controls and validation beads that have been provided by the manufacturer (Spherotech 8-Peak Validation Beads FL1—FL3, BD Accuri).

### Statistical analysis

All data were subjected to normality test using Kolmogorov-Smirnov test and the homogeneity of variances by Cochran C test (Statistica 7.0, StatSoft, Inc). Statistical significance of one and two way ANOVAs were accepted at *P* < 0.05 and post-hoc Tukey test was adopted to determine the source of variation among the molt stages. The data are presented as mean ± SE (n), where n is the number of crabs, unless stated otherwise. For shell-hardness data, analysis of covariance (ANCOVA) with general lineal model (Y = a + bX) was used.

## Results

### Identification of hemocyte types in the hemolymph of *C*. *sapidus*

Three types of hemocytes are found in the hemolymph of *C*. *sapidus* (n = 6 crabs): granulocytes, semigranulocytes and hyaline cells (S4A and S4B Fig). Granulocytes contain a nucleus in the center of their cells and cytoplasmic granules with the average cell diameters of 12.0 ± 1.5 μm (n = 35 cells). Semigranulocytes show the nucleus in the center of the cytoplasm and contain fewer cytoplasmic granules than granulocytes. They are 9.2 ± 1.1 μm (n = 35 cells) and smaller than granulocytes. Hyaline cells have a prominent nucleus in the center of the cytoplasm but without cytoplasmic granules. They are the smallest hemocytes with diameters of 8.5 ± 1.1 μm (n = 35 cells).

### Role of *CasPPO-hemo* in the initial shell-hardness

**Fig 1:**
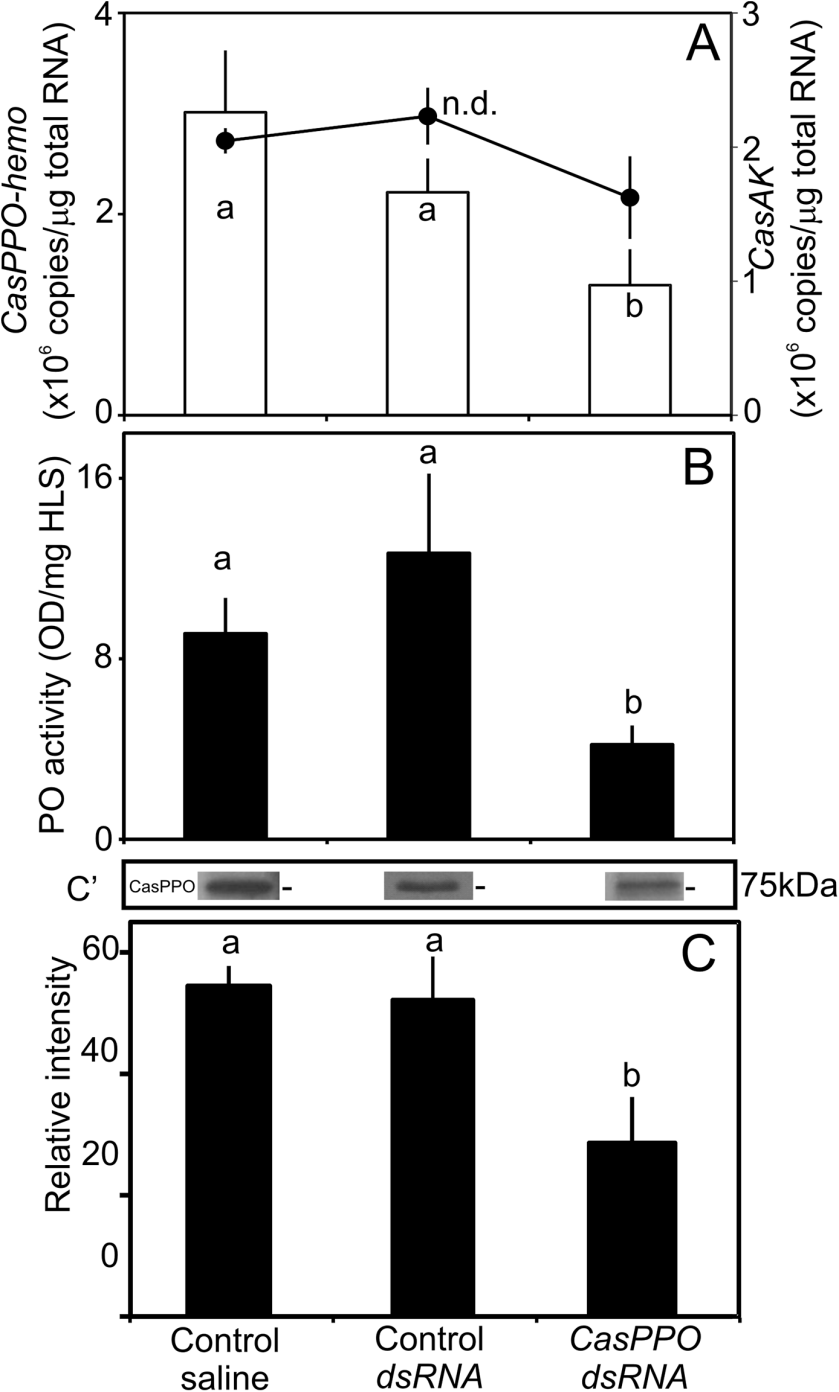
Effects of multiple injections of *CasPPO-hemo-dsRNA* on shell-hardness. Intermolt crabs (n = 7/ group) were injected with 10 μg of *CasPPO-hemo-dsRNA*, control dsRNA, or saline every other day until ecdysis. (A) Levels of *CasPPO-hemo* transcripts, 24 hrs after ecdysis, (bars) in hemocytes (n = 7) and levels of *CasAK* transcirpts (line) in the same cDNA samples that are assayed as a reference gene. Different letters denotes the significant differences at *P* < 0.05. (B) CasHLS-PO activity at postmolt (24 hrs after ecdysis, n = 7). (C) The western blot analysis (c’) identifies the presence of CasPPO-hemo in CasHLS in the samples. n.d. = no statistical difference.

The multiple injections of *CasPPO-hemo*-*dsRNA* reduce the levels of its transcript in hemocytes by ~57% (Fig 1A), compared to controls. The levels of protein in hemocytes are also decreased by ~54% for the CasHLS-PO activity (Fig 1B) and by ~50% for relative immunoreactivity in CasHLS (Fig 1C), compared to controls.

**Fig 2:**
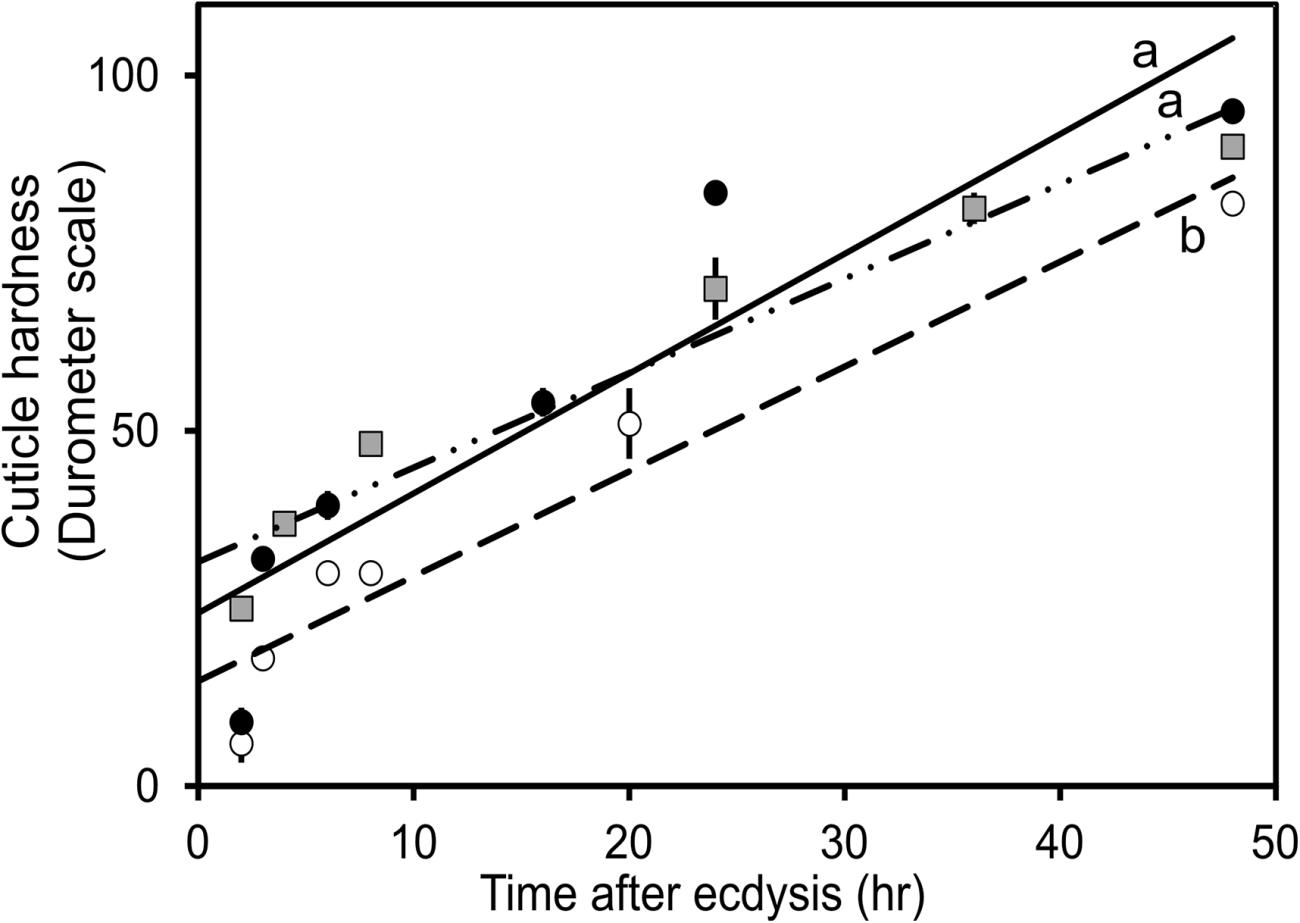
Shell-hardness of the new cuticle during 48 hrs after ecdysis. Intermolt crabs (n = 7) were injected with 10 μg of *CasPPO-hemo-dsRNA* every other day until ecdysis and the hardness of the postmolt cuticle was measured by a hand-held durometer (durometer scale = 0–100 DU). Dashed line = *CasPPO-hemo-dsRNA* injected group (R^2^ = 0.93), solid line = control saline (R^2^ = 0.84), and dashed-dot line = control dsRNA (R^2^ = 0.94). ANCOVA analysis of general linear models (Y = a + bX) shows a significant difference (*P* < 0.05, noted with different letters) in the hardening of the cuticle of *PPO*-*dsRNA* injected and control crabs (n = 7). The data are shown as mean ± SE DU.

For the shell-hardness during 48 hrs after ecdysis, the animals that received the *CasPPO-hemo-dsRNA* show significantly lower than the controls (Fig 2). At the first ~2 hrs after ecdysis, the cuticle hardness of *CasPPO-hemo-dsRNA* injected crabs is measured as18 ± 1 DU (n = 7), which is significantly (*P* < 0.05) lower than that of controls: 32 ± 3 DU (control saline; n = 7) and 25 ± 3 DU (control *dsRNA*; n = 7). At 24 hrs after ecdysis, the *CasPPO-hemo-dsRNA* injected crabs increase in the hardness ~33 DU to 51 ± 2 DU (n = 7), while the increment of controls is ~50 DU, resulting in the hardness of 84 ± 5 DU (control saline; n = 7) and 70 ± 3 DU (control *dsRNA*; n = 7). The controls have markedly higher values of DU (*P* < 0.05) than those of the *CasPPO-hemo-dsRNA* injected crabs. At 48 hrs after ecdysis, the carapace of the *CasPPO-hemo-dsRNA* injected animals continues to increase in the hardness of ~31 DU to 82 ± 2 (n = 7), although they are still slightly lower than those of controls with 95 ± 2 DU (control saline; n = 7) and 90 ± 2 DU (control *dsRNA*; n = 7).

Fig 2 shows the values of HD_50_ (time after ecdysis when the cuticle reach the 50% of hardness). The HD_50_ values of *CasPPO-hemo*-*dsRNA* group are 18.9 ± 1.4 hrs (n = 7) which is significantly (*P* < 0.05) higher than the values of controls: 8.7 ± 0.9 hrs (control saline; n = 7) and 7.1 ± 0.8 hrs (control *dsRNA*; n = 7).

**Fig 3:**
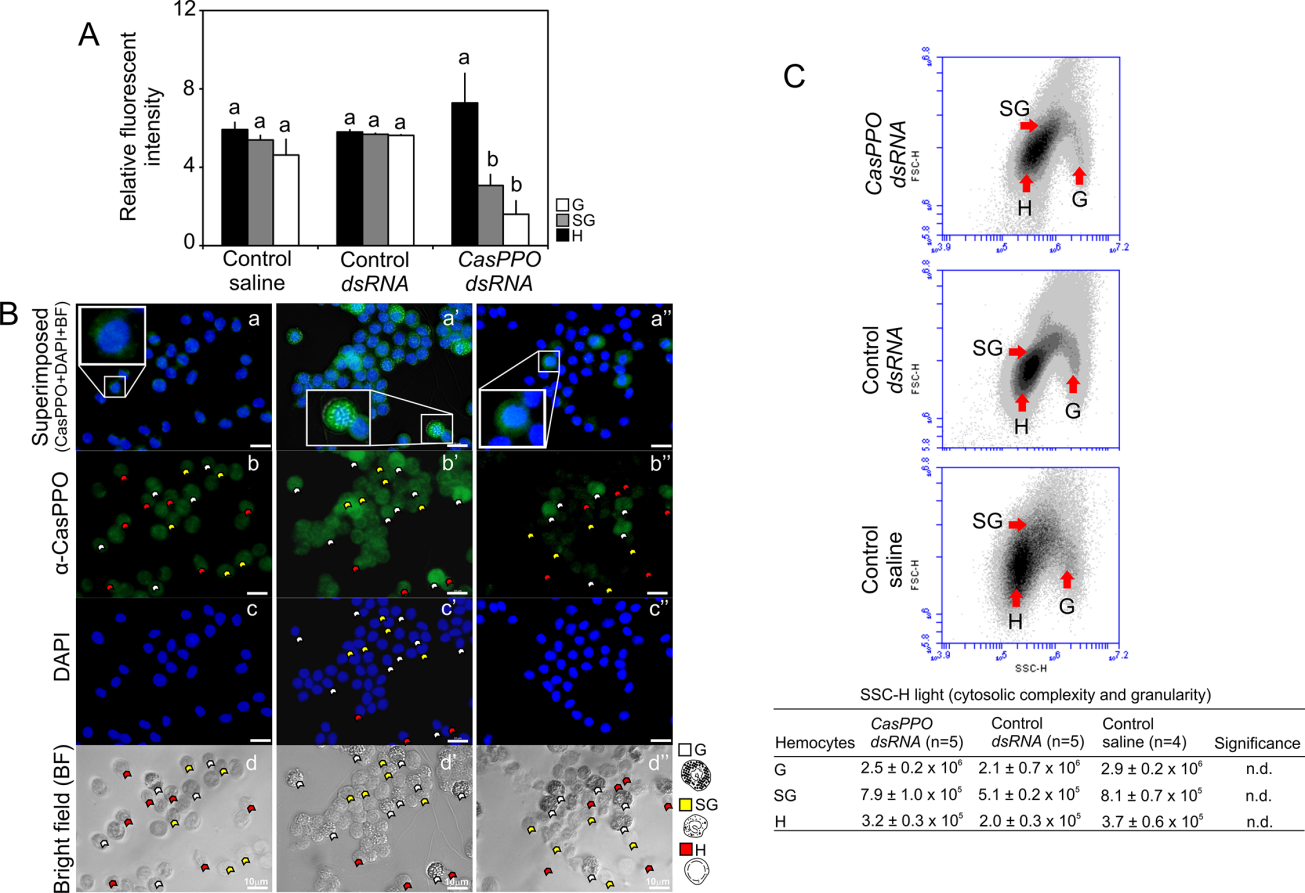
Levels of CasPPO-hemo in hemocytes after multiple injections of *CasPPO-hemo-dsRNA*. (A) Fluorescent intensity analysis, (B) ICC (left to right = control saline, control *dsRNA*, *CasPPO-hemo dsRNA*), and (C) flow cytometry analysis of hemocytes from *CasPPO-hemo-dsRNA* and control groups. The positive α-CasPPO-hemo immnunopositve staining of the cells obtained at 24 hrs after ecydsis was determined for fluorescent intensity, which was calculated using ImageJ. The insertion in the ICC pictures shows a positive hyaline cell (a) and granulocyte (a’ and a”). The flow cytometry indicates no changes in the cytosolic complexity and granularity of hemocytes in the *CasPPO-hemo*-*dsRNA* injected group. The values of cytosolic complexity (SSC-H) are shown as mean ± SE (n = 4–5). Different letters indicate significant differences at *P* < 0.05: B and C) Two way ANOVA. n.d. = no significant differences. Bar sacle = 10 μm. G = granulocytes; SG = semigranulocytes; H = hyaline cells.

All the hemocytes obtained from controls and *CasPPO-hemo-dsRNA* injected animals at 24 hrs after ecdysis are present with PPO (Fig 3A and 3B). The hemocytes from *CasPPO- hemo-dsRNA* injected animals display significantly reduced immunostaining intensities with α-CasPPO-hemo. Specifically, semigranulocytes and hyaline cells have reduced immunostaining intensities, compared to those of controls (Fig 3A and 3B). However, there are no differences (*P* > 0.05) in cytosolic complexity and granularity between all the hemocytes of *dsRNA* injected and control crabs (Fig 3C).

Both *CasPPO-hemo-dsRNA* and control groups show no significant differences in the size and number of the hemocytes and molt increment and intervals (S5 Fig; S1 Table).

### Differential hemocyte counting (DHC) during the molt cycle

**Fig 4:**
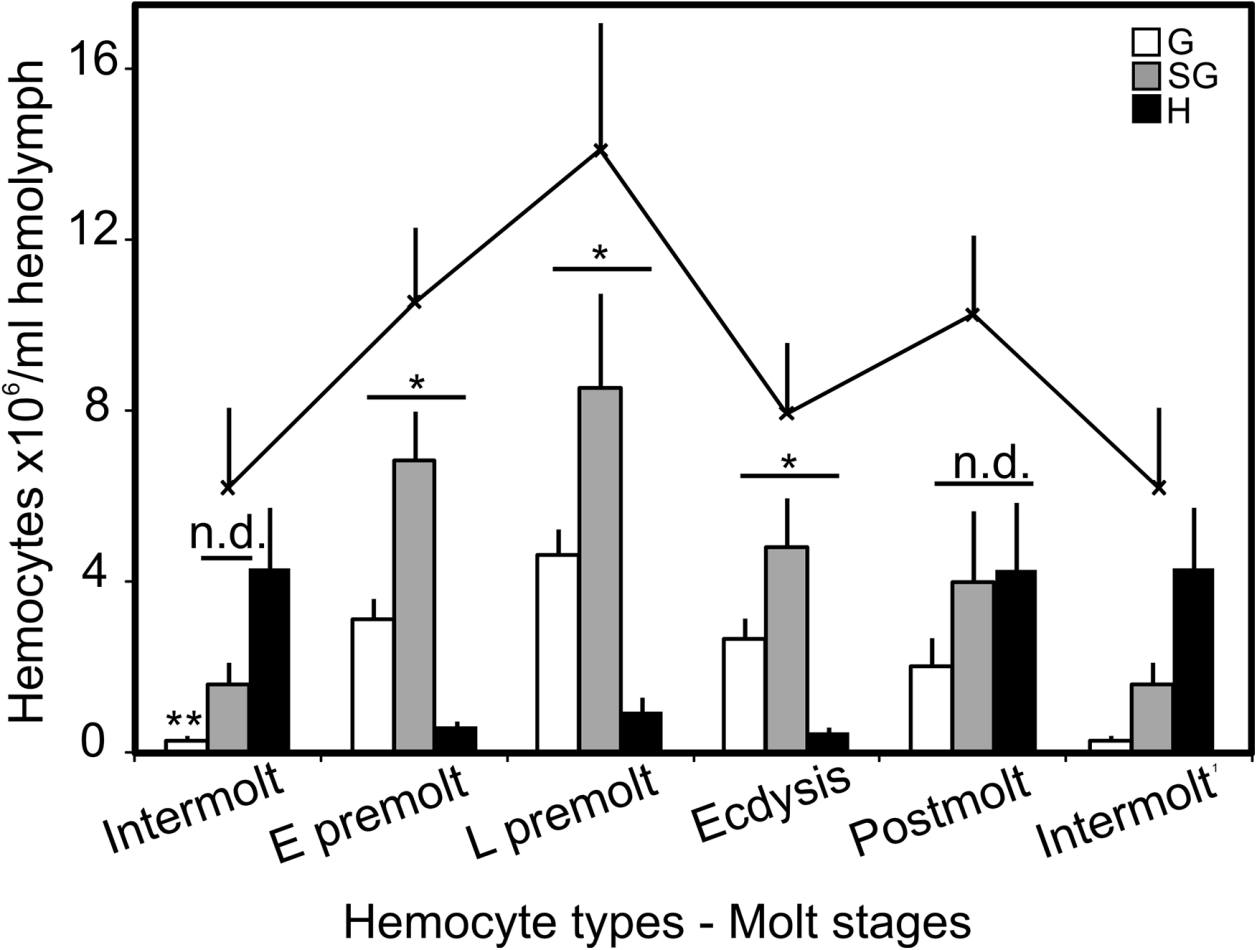
Differential hemocyte counting (DHC) during the molt cycle of *C*. *sapidus* using the flow cytometry analysis. The values of THC (solid line) do not significantly differ among the different molt stages (n = 18). Each bar represent a hemocyte type. n.d. = no difference; the ‘*’ and ‘**’ denote significant difference at *P* < 0.05 and *P* < 0.01 (two way ANOVA), respectively. G = granulocytes; SG = semigranulocytes; H = hyaline cells.

Throughout the molt cycle, *Callinectes* hemolymph always carries three types of hemocytes. The number of each cell types (DHC) varies significantly during the molt cycle, whereas the THC changes modestly. At intermolt, the THC ranges 6.2 ± 1.9 x10^6^ cells/ml hemolymph (n = 6) and increases up to 14.1 ± 3.0 x10^6^ cells/ml hemolymph (n = 6) at late premolt. At intermolt, hyaline cells are the majority of hemocytes (Fig 4 and Table 3) with 4.3 ± 1.4 x10^6^ cells/ml hemolymph (69.9 ± 22.6% of THC). Semigranulocytes are the second major type with 1.6 ± 0.5 x10^6^ cells/ml hemolymph (25.8 ± 7.9% of THC) and granulocytes are least with 0.3 ± 0.1 x10^6^ cell/ml hemolymph (4.2 ± 2.0% of THC).

**Table 3:** Changes in different cell types in hemocyte populations of the hemolymph of *C*. *sapidus* during the molt cycle.

	Intermolt	E. premolt	L. premolt	Ecdysis	Postmolt
Hs	69.9 ± 22.6^a^	5.9 ± 1.2^a^	6.8 ± 2.3^a^	6.0 ± 1.3^a^	41.4 ± 15.5^a^
SGs	25.8 ± 7.9^a^	64.7 ± 10.9^b^	60.5 ± 15.8^b^	60.7 ± 14.4^b^	39.0 ± 15.0^a^
Gs	4.2 ± 2.0^b^	29.4 ± 4.9^c^	32.8 ± 4.4^c^	33.4 ± 6.3^c^	19.6 ± 6.7^a^

Relative abundance (Mean ± SE %, Intermolt, Late premolt and Postmolt: n = 6; Early premolt: n = 18, Ecdysis: n = 7). Hs = hyaline cells; SGs = semigranulocytes; Gs = granulocytes. Statistical significance at *P*<0.05 is determined using two-way ANOVA and is noted with different letters.

The animals at early premolt and late premolt contain 11.0 ± 1.7 x10^6^ cells/ml hemolymph and 14.0 ± 3.0 x10^6^ cells/ml hemolymph, respectively. The composition of hemocyte types at early and late premolt in the hemolymph changes, compared to that at intermolt. Specifically, semigranulocytes and granulocytes become the major types of hemocytes. At early premolt, the numbers of semigranulocytes and granulocytes are increased significantly to 6.8 ± 1.2 x10^6^ cells/ml hemolymph (64.7 ± 10.9% of THC) and 3.1 ± 0.5 x10^6^ cells/ml hemolymph (29.4 ± 4.9% of THC), respectively. At late premolt, the numbers of semigranulocytes are 8.5 ± 2.2 x10^6^ cells/ml hemolymph (60.5 ± 15.8% of THC) and those of granulocytes are 4.6 ± 0.6 x10^6^ cells/ml hemolymph (32.8 ± 4.4% of THC). Hyaline cells are counted only with 0.6 ± 0.1 x10^6^ cells/ml hemolymph (5.9 ± 1.2% of THC) at early premolt and 1.0 ± 0.3 x10^6^ cells/ml hemolymph (6.8 ± 2.3% of THC) at late premolt.

At ecdysis, THC are reduced to 7.9 ± 1.7 x10^6^ cells/ml hemolymph, but the composition of hemocyte types remains the same as those at early and late premolt stages. At ecdysis stage, semigranulocyte numbers are reduced to 4.8 ± 1.1 x10^6^ cells/ml hemolymph (60.7 ± 14.4% of THC) while granulocytes are counted to 2.6 ± 0.5 x10^6^ cells/ml hemolymph (33.4 ± 6.3% of THC). The values of hyaline cells are 0.5 ± 0.1 x10^6^ cells/ml hemolymph, which equals 6.0 ± 1.3% of THC.

At postmolt, THC consists of all three hemocyte types at close contribution (Fig 4). The number of hyaline cells significantly increases to 4.2 ± 1.6 x10^6^ cells/ml hemolymph (41.4 ± 15.5% of THC, *P* <0.05), compared to that at ecdysis. Semigranulocytes are constant in their numbers, although significantly lower (39.0 ± 15% of THC, *P* < 0.05), than those at premolt. Granulocytes are 2.0 ± 0.7 x10^6^ cells/ml hemolymph, representing 19.6 ± 6.7% of THC.

### Immnunostaining of CasPPO-hemo in each type of hemocytes during the molt cycle

**Fig 5:**
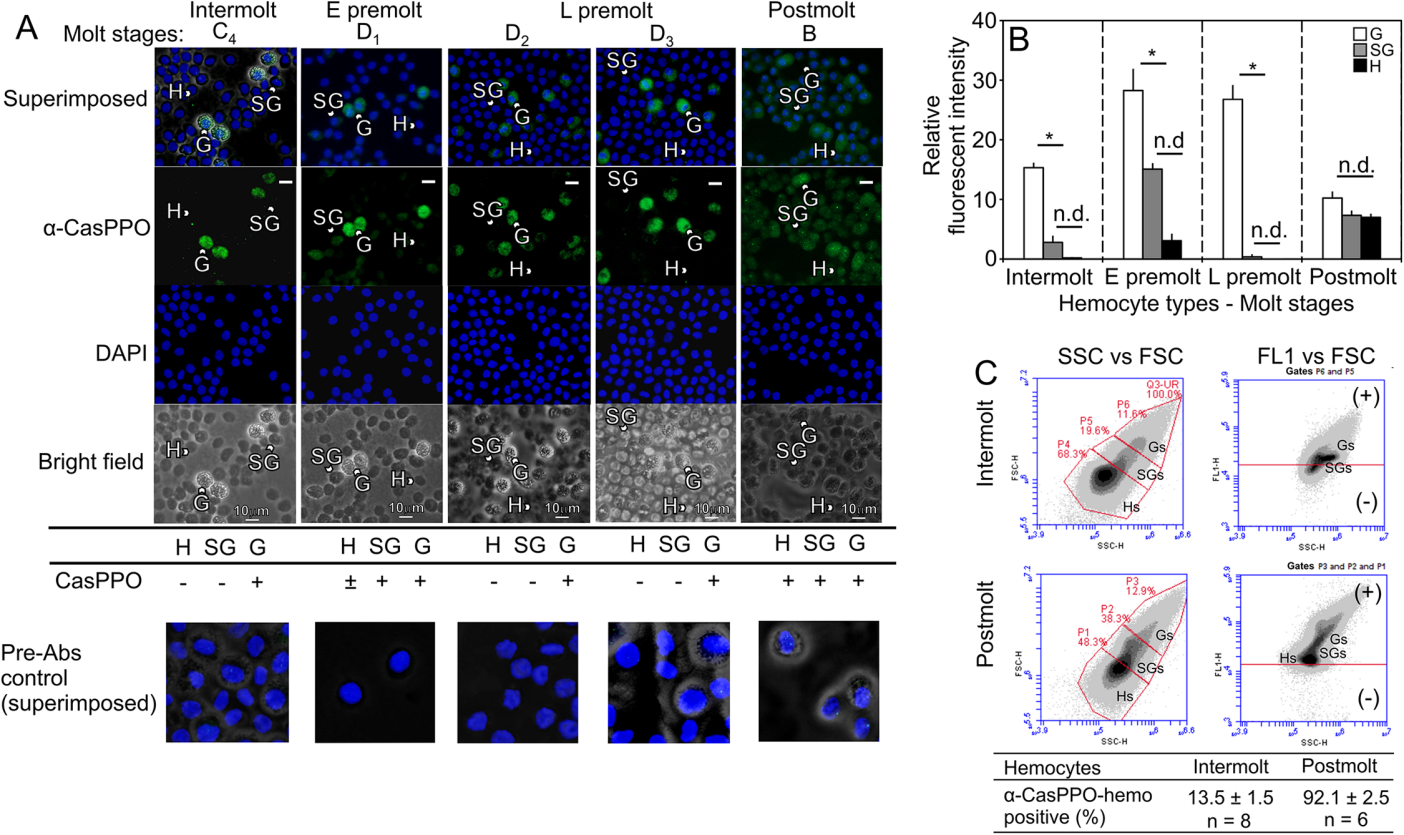
Identification of CasPPO-hemo in hemocytes during the molt cycle of *C*. *sapidus*. Immunostaining of hemocytes at different molt stages using a specific α-CasPPO-hemo. Granulocytes contain PPO during all the molt stages, while semigranulocytes and hyaline cells show a PPO protein expression which is molt-stage dependent. Signals: + PPO and–PPO. (A) ICC (n = 5–6) and (B) fluorescent intensity of the positive signal of α-CasPPO-hemo staining using ImageJ. (C) Immunostaining of CasPPO-hemo by flow cytometry analysis: At intermolt stage, ~14% of hemocytes are PPO positive, while at postmolt, the PPO positive hemocytes are over 90% (n = 5–8). Bar scale = 10 μm. G = granulocytes; SG = semigranulocytes; H = hyaline cells.

The presence of CasPPO-hemo protein in hemocytes is determined using ICC and flow cytometry. ICC results show that granulocytes display the strongest α-CasPPO-hemo immunostaining consistently through molt cycle (Fig 5A and 5B; n = 5–6). Hyaline cells exhibit α-CasPPO-hemo immunostaining only at postmolt stage (Fig 5A and 5B; n = 5–6), while semigranulocytes are immunosensitive only at early premolt and postmolt stages.

The flow cytometry analysis (Fig 4C; n = 6–8) of α-CasPPO-hemo immunostained hemocytes is in agreement with that of ICC. At intermolt, the 13.5 ± 1.5% of the cells are positive stained with α-CasPPO-hemo, while during postmolt over 90% of the hemocytes are immunopositive to α-CasPPO-hemo.

## Discussion

This study provides evidence that PPO present in hemocytes is an essential component in the shell-hardness of *C sapidus*. During the molt cycle, PPO is expressed in different types of hemocytes in the hemolymph of *C*. *sapidus*. At postmolt, semigranulocytes and hyaline cells are the major cellular sources of PPO, while at intermolt, granulocytes produce PPO.

It appears that decapod crustaceans possess three types of hemocytes. The hemolymph of *C*. *sapidus* always carries three different kinds of hemocytes throughout the molt cycle, as is similar to those found in other crustacean species including *H*. *americanus*, *P*. *interruptus*, *L*. *grandis*, *P*. *leniusculus*, *P*. *japonicus*, *P*. *clarkii*, *U*. *pugilator* and *S*. *ingentis* [31,36,37,39–41].

The role of CasPPO-hemo in the shell-hardening process in *C*. *sapidus* has been examined with multiple injections of *CasPPO-hemo-dsRNA*. This RNAi approach is employed successfully to define the functions of a gene of interest in several crustacean species [50,56,57]. The *CasPPO-hemo-dsRNA* injected crabs show significantly and specifically reduced levels of *CasPPO-hemo* transcripts and CasPPO-hemo protein, subsequently resulting in low PO activity, compared to saline and control *dsRNA* injected ones. More importantly, the animals treated with *CasPPO-hemo-dsRNA* have a significantly slower the shell-hardening process than control groups.

Since the hemolymph of *C*. *sapidus* contains three types of hemocytes [38,58], the following question was asked: Which hemocyte type(s) that produce PPO is/are affected by *dsRNA* injections at postmolt? In this study, the values of THC that have been re-examined using flow cytometry are in agreement with the levels obtained by cell counting using a hemocytometer [33].

CasPPO antiserum that is generated against two regions located between the hemocyanin N and M domains and between hemocyanin M and C domains of CasPPO [33] recognizes ~0.6 μg HLS protein (= ~900 hemocytes equivalent) at a final dilution of 1:2000 (S2 Fig). The pre-absorption controls show no staining in the hemocytes at the molt stages examined (Fig 5A).

Multiple injections of *CasPPO-hemo-dsRNA* affect the immunostaining intensities of hemocytes at postmolt. Both semigranulocytes and hyaline cells show the most significantly reduced CasPPO-hemo immunostaining intensities by 19% and 65%, respectively. In the granulocytes, there are no significant differences in the staining intensities nor the granularity, compared to those of the controls.

It seems that the hemocytes undergo inherent changes in their functions during the molt cycle (Fig 5B) and that different cell types may contribute PPO activity at a given molt stage. At postmolt, all three types of hemocytes are α-CasPPO-hemo positive. Interestingly, hyaline cells exhibit positively PPO immunostained only at postmolt, suggesting that there may be a particular function for these hemocytes after ecdysis. This notion is supported by another observation made in *U*. *pugilator* [31]. In *U*. *pugilator*, hyaline cells that are found with PPO positive during ecdysis also implicate the role of these hemocytes in the shell-hardening process. Together with our data on the reduced PPO contents in semigranulocytes and hyaline cells by the injection of *dsRNA* that slow shell-hardening process of *C*. *sapidus* at postmolt, we propose that hyaline cells and semigranulocytes may indeed play a direct role in this process of decapod crustaceans.

Our data also implicate that different types of hemocytes may be recruited into the hemolymph at a molt-stage specific manner. It also infers that hematopoietic activity may inherently differ by molt stage. Interestingly, a molt stage-dependent pattern of hemocyte production is shown in the crustacean hematopoietic organs, together with increasing the mitosis at premolt [59] primarily for the release of granulocytes. Within the hematopoietic tubules, the hemocytes are being differentiation into a specific type of cells such as granulocytes and semigranulocytes [60–64]. However, functional differentiation of these hemocytes by expressing PPO is completed by their release into the hemolymph [40,61]. More specifically, the contribution of each type of hemocytes toward total PPO is different by a molt stage, which indicates that the hematopoiesis involves a molt stage-specific hemocyte differentiation. Further study is needed, specifically isolating the naïve differentiated hemocytes from the hematopoietic tubules and recognizing newly released hemocytes using fbromodeoxyuridine, BrdU [61]).

It remains for further studies to determine what regulates the hematopoietic activities of decapod crustaceans during the molt cycle. The molting hormone, ecdysteroid that usually stimulate cellular mitotic activities in the various tissues at premolt [4,65–67] may also affect the activity of hematopoietic organs. Small cell signaling molecules like astakines play a role in hemocyte proliferation [68,69]. Specifically, astakines 1, an invertebrate cytokine expressed in the hematopoietic organs and hemocytes, promotes the proliferation and differentiation of hemocytes in *P*. *leniusculus* [68,69].

Our data provide evidence of the involvement of hemocytes and hemocyte PPO in the shell-hardening process. However, a question remains of how hemocyte PPO is transported into the new cuticle for the hardening process. While hemocyte tissue infiltration is reported in other crustaceans [70,71], it is also reported in an insect species that PPO is transported from hemocytes into the cuticle through the hypodermis [34,35]. Additionally, arthropod tanning hormone bursicon is implicated in recruiting a particular type of heavily granulated hemocytes beneath the hypodermis at ecdysis [42]. On the other hand, hyaline cells containing PPO may pass easily through the hypodermis to rapidly distribute the PPO into the new cuticle and quickly initiate the shell-hardening during postmolt.

Some differences are found with two different counting methods, between ICC and flow cytometry, for the proportional contribution of PPO cell type. Specifically, the differentiation of hemocyte types by flow cytometry is based on internal complexity (SSC-H) and size (FSC-H) of the cells. Therefore, the numbers of granulocytes and semigranulocytes might have been somewhat under- or over-estimated. Semigranulocytes have been found PPO positive at the intermolt of other crustacean species [36,37,39–41]. On the other hand, the ICC shows granulocytes as the PPO positive hemocytes at the intermolt stage of *C*. *sapidus*, implying that the flow cytometry analysis could underestimate the numbers of granulocytes.

Differences in levels of immunostaining detected in the same type of hemocytes, specifically granulocytes, during the molt cycle, may indicate differential translation rates of PPO. Little is known about the translation rate of *CasPPO-hemo* or the half-life of the protein. However, levels of *CasPPO-hemo* transcripts remain constant during the molt cycle [33]. When comparing the amounts of CasPPO-hemo in a single granulocyte at intermolt and postmolt, there are significant differences in the levels of CasPPO-hemo and PPO activity. For example, at intermolt, ~2.5 x 10^5^ granulocytes/ml hemolymph are accounted for expressing ~3.0 x 10^5^ transcripts of *CasPPO*-hemo [33]. If it is assumed that each granulocyte contains the same amounts of *CasPPO-hemo* transcripts, a single granulocyte contains 1.2 transpcripts (equiv. PO activity ~6.5 x 10^−6^ OD/mg HLS/cell [33]). At postmolt, all cell types express *CasPPO-hemo* transcripts. At this stage, 2.0 x 10^6^ granulocytes are accounted for expressing 1.7 x 10^4^ transcripts, with a single granulocyte containing 0.009 transcripts (equiv. PO activity ~1.7 x 10^−7^ OD/mg HLS/cell). While it is not certain if granulocytes at intermolt are exactly the same cells as those found at postmolt, it is apparent that the same type of cells contains different levels of *CasPPO-hemo* transcripts and PO activity.

**Fig 6:**
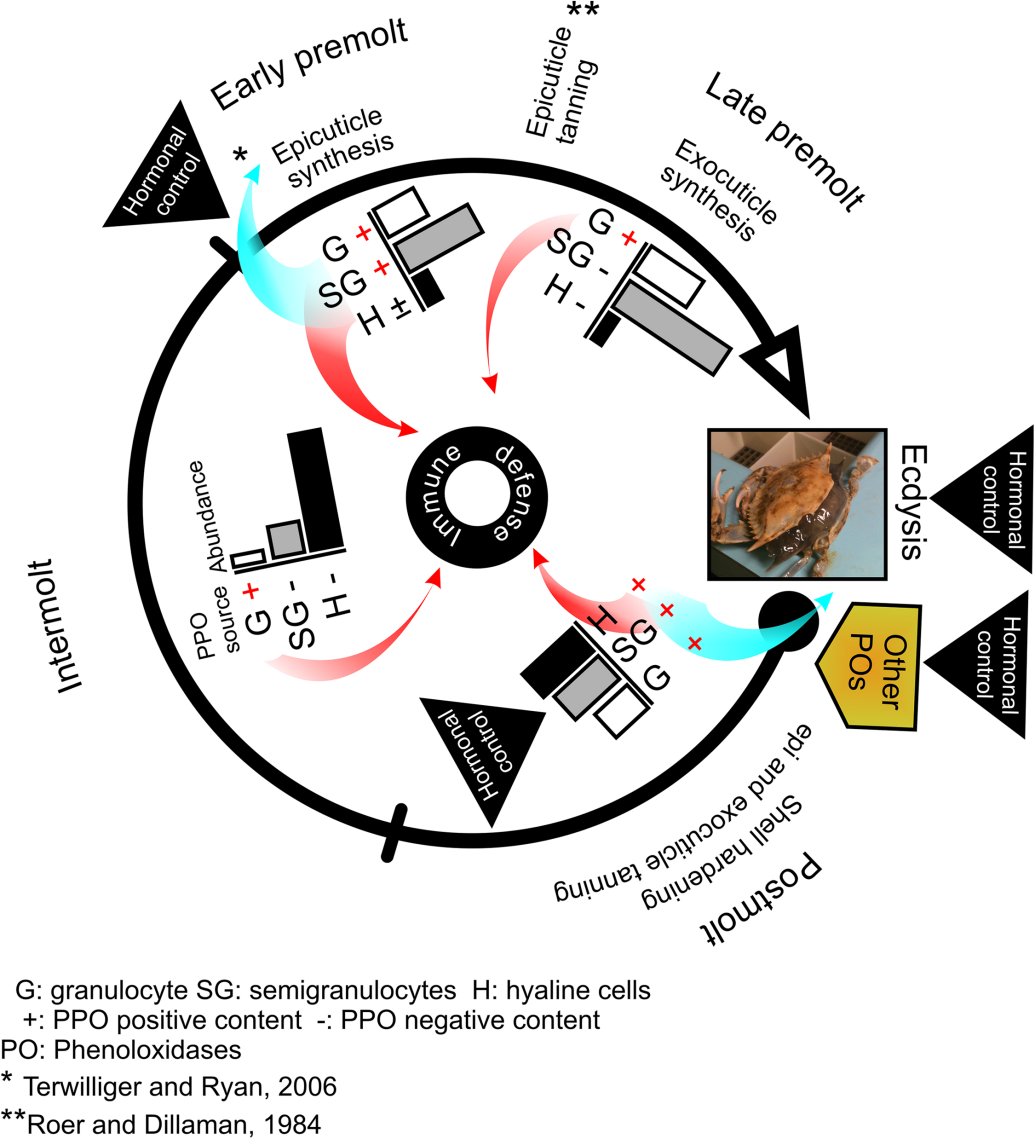
A proposed model for the involvement of hemocytes and its PPO (CasPPO-hemo) in the shell-hardening process of the blue crab, *C*. *sapidus*. The diagram summarizes the results obtained from the current study. Changes in hemocyte numbers are shown with bar plots. The positive (+) and negative (-) symbols indicate the presence or absence of PPO expression in each type of hemocytes, respectively. Blue arrows indicate the potential transport of PPO from hemocytes into the cuticle, as described [38]. Red arrows indicate the specific hemocytes and PPO role in the immunity system [40,41]. Asterisks denote that the studies were carried out by other researchers.

Taken all together, a proposed model is presented that integrates hemocytes and CasPPO-hemo in the shell-hardening process (Fig 6). Semigranulocytes and granulocytes are estimated as the main hemocyte types at early and late premolt stages. At premolt, the synthesis and layering of the new soft cuticle [2,8] is induced by the increase in the levels of hemolymphatic ecdysteoids [4,5]. The positive detection of PPO in semigranulocytes and granulocytes at early premolt may be associated with the layering the new cuticle through the PPO transport as suggested [32]. Moreover, it is known that more than one *PPO* gene exist in insects [29,72–75]. While only one *PPO* cDNA has been reported in some decapod crustacean species [33,76–78], multiple PPO sequences reported in several shrimp species including *Penaeus monodon* and *Macrobrachium rosenbergii*, *Litopenaeus vannamei*, *Fenneropenaeus chinensis*, and *Marsupenaeus japonicus* [79–84] may have been derived from separate *PPO* genes. PO activity contibuted by the hemocytes might not be sufficient for the process; hypodermis may express its PO(s) (PPO and/or laccase) to assist the cuticle sclerotization at a specific time during the molt cycle, together with hepatopancreas and hindgut that are known to produce specific PPOs for the immunity of crustaceans and insects [81,85]. It is plausible that *C*. *sapidus* may contain more than one *PPO* cDNA and gene as reported in shrimp species [79–82].

Laccase is a key enzyme for the hardening process of some insects [10,11,22,86–88]. In crustaceans, the presence of a laccase or its role in shell-hardening process has not yet been described to date. Given the difference in substrate specificity of PPO and laccase [11,22], however, the involvement of each of these enzymes in shell-hardening may not be the same. Hypodermis, the tissue that may likely be essential for the conduit of hemocyte PPO transport into the new cuticle, also expresses the PPO activating enzymes [89,90] for the PPO activation. The sclerotization occurring through the first two hours after ecdysis involves specific types of hemocytes [42]. PPO expressed in the hyaline cells and semigranulocytes may likely be involved in the shell-hardening process at the postmolt. Granulocytes, as the constant PPO reservoir throughout the molt cycle play a role in innate immunity [28].

## Supporting Information

S1 Fig Saturated ammonium sulfate (SAS) precipitations of proteins present in hemocyte lysates (HLS) and its PO activity isolation. (A) The PO activity was recovered in % SAS. (B) The Identification of CasPPO-hemo in the SAS fractions was determined using western blot analysis with a rabbit CasPPO-hemo antiserum (α-CasPPO-hemo).
(TIF)

S2 Fig Characterization of α-CasPPO-hemo. The properties (sensitivity and a dilution factor) of α-CasPPO-hemo are determined using western blot analysis with HLS_30%SAS_. Western blot analysis using the α-CasPPO-hemo (A) at 1:2000 dilution and (B) several different dilutions. Pre-Abs = pre-absorption control. For Pre-Abs, synthetic CasPPO peptides (10 nmol each) were incubated with α-CasPPO-hemo (a final dilution at 1:10) for overnight at 4°C through gentle mixing on a magnetic stir. It was then diluted at a final dilution of 1:2000, same as α-CasPPO-hemo.
(TIF)

S3 Fig Sampling areas of the shell hardness during postmolt. The hardness is measured on 6 spots (white dots) located on the dorsal carapace mesogastric area of the blue crabs (between dashed lines) using a durometer. The blue crab image is downloaded from the symbol library (http://ian.umces.edu/symbols/).
(TIF)

S4 Fig Differentiation of Hemocyte types. The blue crab hemocytes are A) microscopically differentiated (H&E = hematoxylin and eosin staining) and B) flow cytometry. The hemocytes are distinguished based on the criteria as described [37,38]. Three main types of hemocytes are recognized in the hemolymph of the animals during the molt cycle: Granulocytes (G), semigranulocytes (SG) and hyaline cells (H). The hemolymph samples are collected in a fixative and read with a flow cytometer.
(TIF)

S5 Fig *CasPPO-hemo* knockdown experiment: Molt increment (the body size) and interval and size of hemocytes in control saline and experimental crabs. Injections of 10 μg of *CasPPO-*hemo-*dsRNA* do not affect (A) molt increments: White bars = time zero; black bars = at ~48 hrs after ecdysis); (B) The molt intervals; and (C) the size of hemocytes in the *dsRNA* injected crabs and control saline group. Open bars = *dsRNA* injected crabs; solid bars = control saline crabs. G = granulocytes; SG = semigranulocytes; and, H = hyaline cells.
(TIF)

S6 Fig *CasPPO-hemo* knockdown time-point using *dsRNA*. The intermolt crabs received 10 μg of CasPPO-hemo-dsRNA every other day. *CasPPO-hemo* transcripts are reduced by ~90% after the second injection and ~60% after 4 injections, compared to controls that received crustacean saline.
(TIF)

S1 Table Hemocyte abundance (cells/ml hemolymph) 24 hrs after ecdysis in *CasPPO-hemo-dsRNA* injected and control crabs. No significant differences (*P* > 0.05) are observed using two way ANOVA.
(PDF)

S1 Protocol Isolation of HLS-PO activity and characterization of anti-CasPPO-hemo (α-CasPPO-hemo). (PDF)
