# Supplementary figures and images for: The Involvement of Hemocyte Prophenoloxidase in the Shell-Hardening Process of the Blue Crab, Callinectes sapidus
Source: PLoS One. 2015 Sep 22;10(9):e0136916. doi: 10.1371/journal.pone.0136916 (PMC4634603; doi:10.1371/journal.pone.0136916)

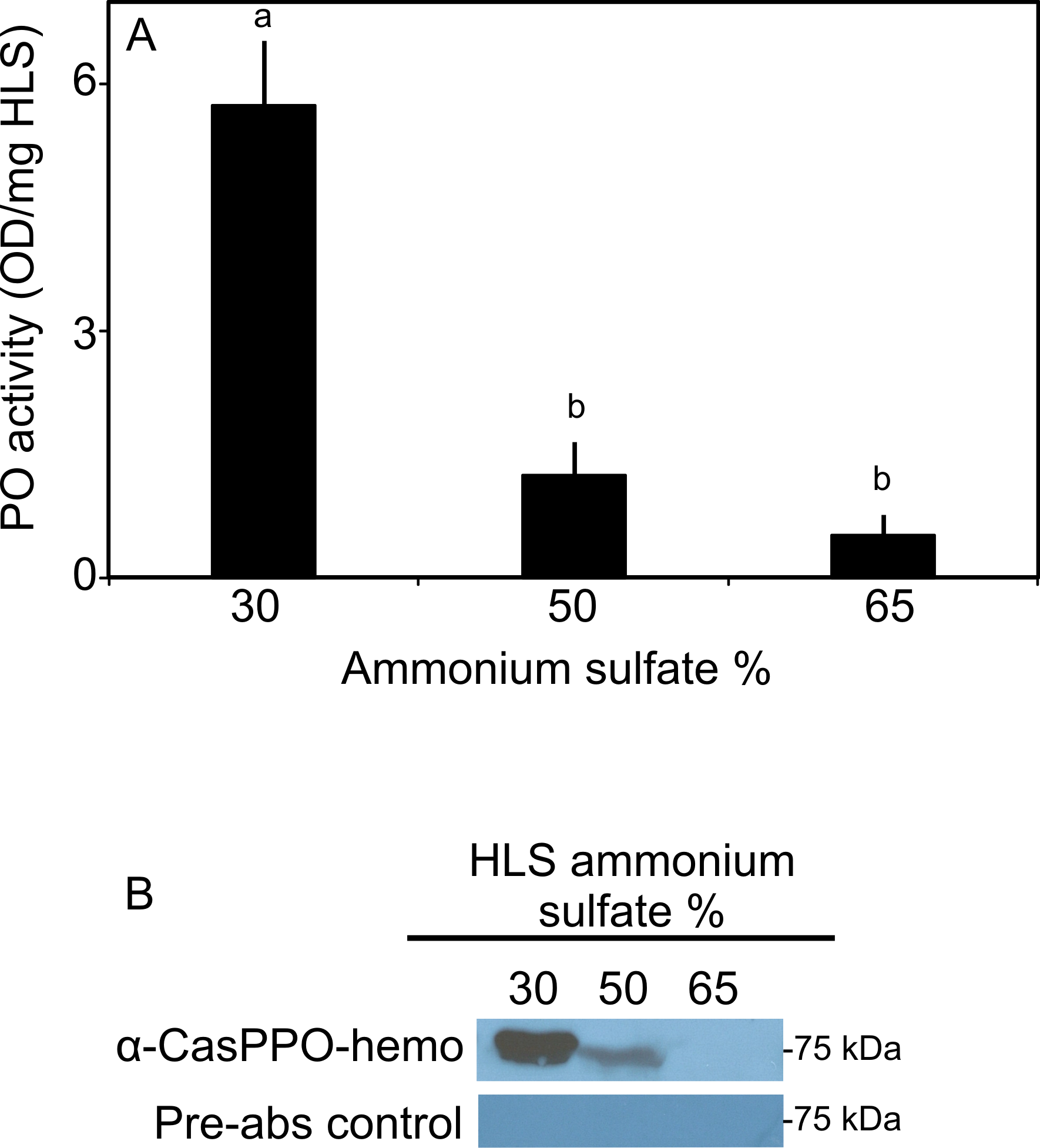

Supplement: S1 Fig — (A) The PO activity was recovered in % SAS. (B) The Identification of CasPPO-hemo in the SAS fractions was determined using western blot analysis with a rabbit CasPPO-hemo antiserum (α-CasPPO-hemo). (TIF) [file pone.0136916.s001.TIF]

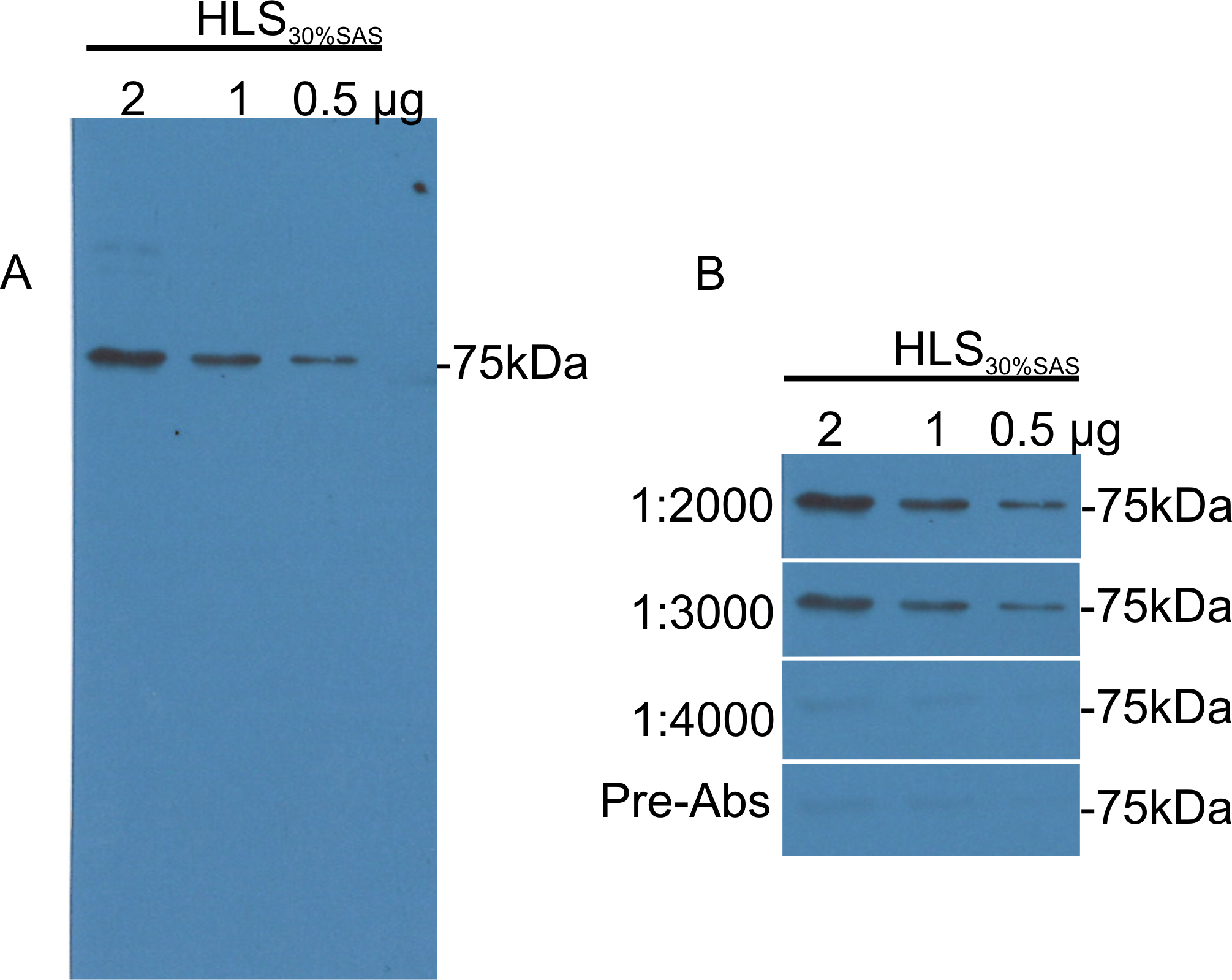

Supplement: S2 Fig — The properties (sensitivity and a dilution factor) of α-CasPPO-hemo are determined using western blot analysis with HLS30%SAS. Western blot analysis using the α-CasPPO-hemo (A) at 1:2000 dilution and (B) several different dilutions. Pre-Abs = pre-absorption control. For Pre-Abs, synthetic CasPPO peptides (10 nmol each) were incubated with α-CasPPO-hemo (a final dilution at 1:10) for overnight at 4°C through gentle mixing on a magnetic stir. It was then diluted at a final dilution of 1:2000, same as α-CasPPO-hemo. (TIF) [file pone.0136916.s002.TIF]

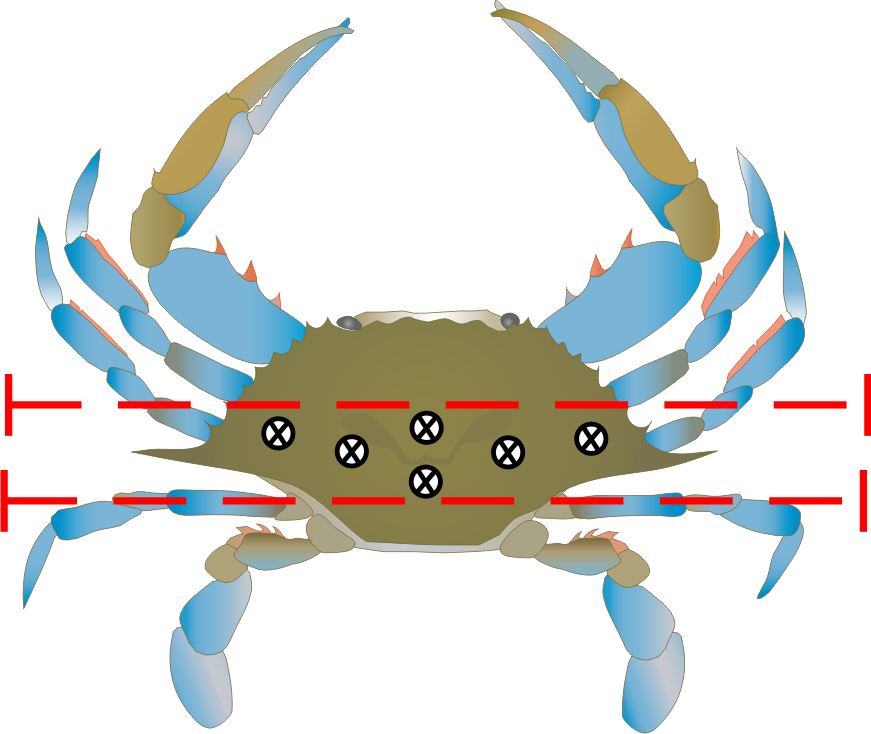

Supplement: S3 Fig — The hardness is measured on 6 spots (white dots) located on the dorsal carapace mesogastric area of the blue crabs (between dashed lines) using a durometer. The blue crab image is downloaded from the symbol library (http://ian.umces.edu/symbols/). (TIF) [file pone.0136916.s003.TIF]

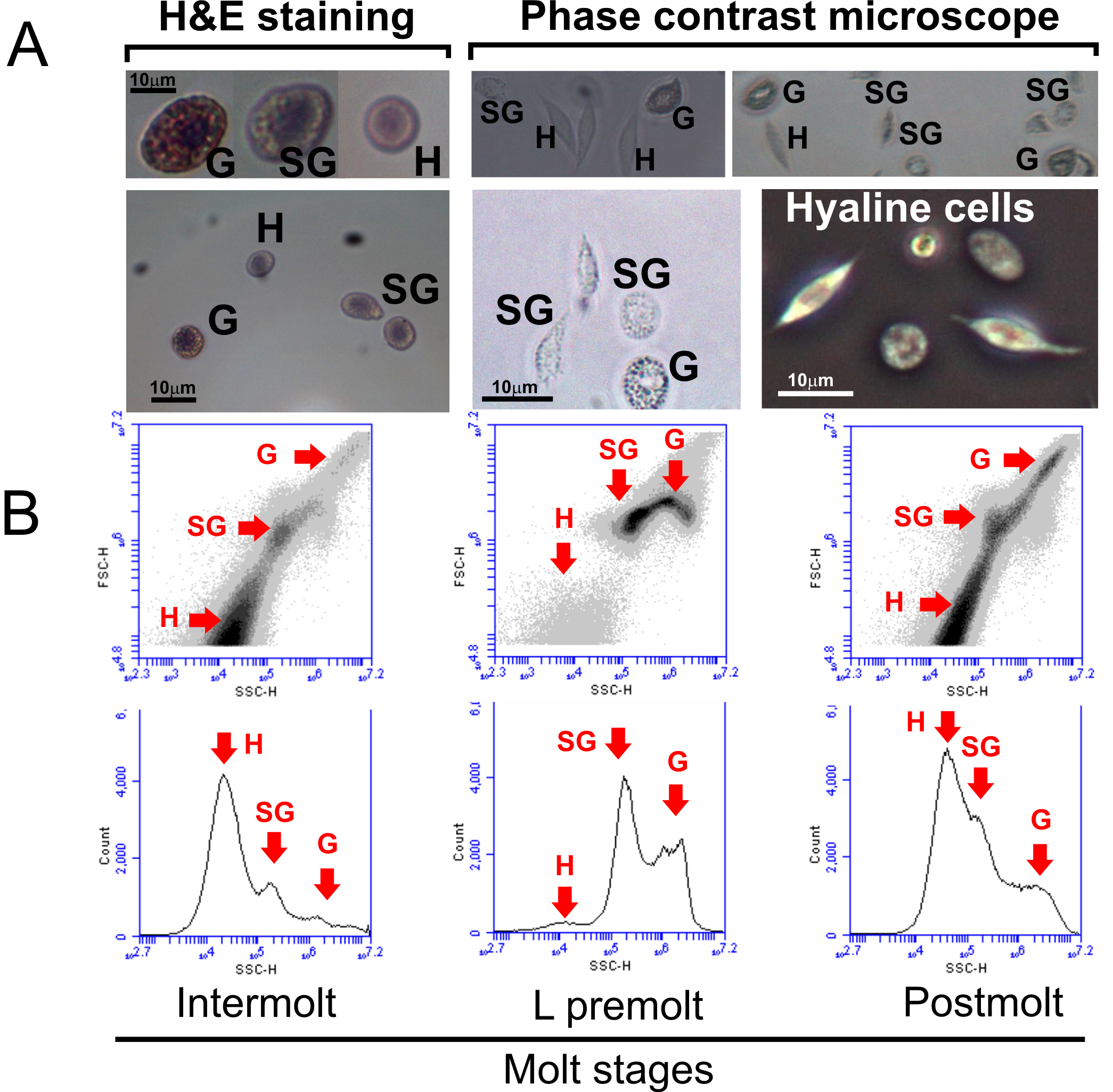

Supplement: S4 Fig — The blue crab hemocytes are A) microscopically differentiated (H&E = hematoxylin and eosin staining) and B) flow cytometry. The hemocytes are distinguished based on the criteria as described [37,38]. Three main types of hemocytes are recognized in the hemolymph of the animals during the molt cycle: Granulocytes (G), semigranulocytes (SG) and hyaline cells (H). The hemolymph samples are collected in a fixative and read with a flow cytometer. (TIF) [file pone.0136916.s004.TIF]

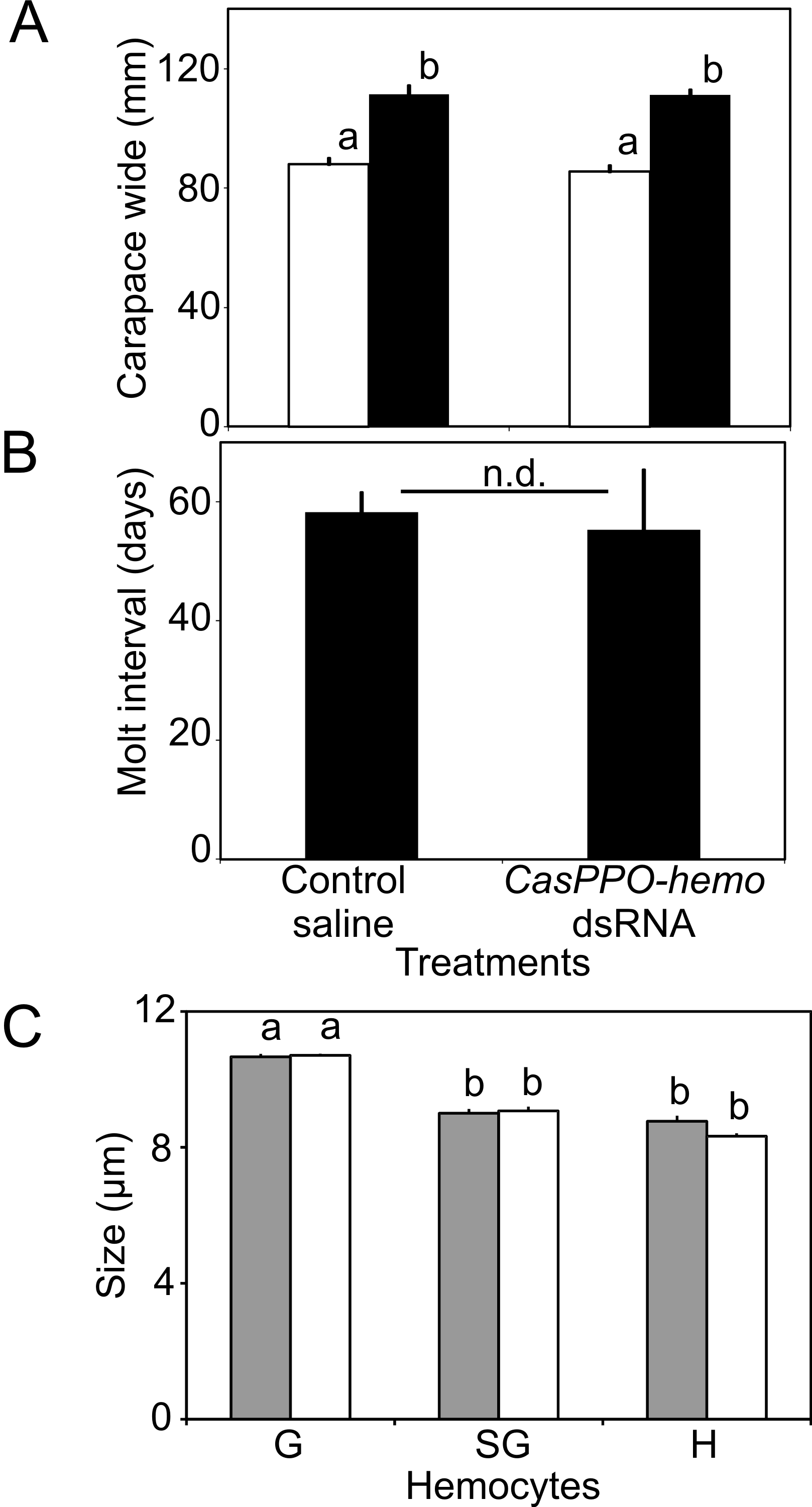

Supplement: S5 Fig — Injections of 10 μg of CasPPO-hemo-dsRNA do not affect (A) molt increments: White bars = time zero; black bars = at ~48 hrs after ecdysis); (B) The molt intervals; and (C) the size of hemocytes in the dsRNA injected crabs and control saline group. Open bars = dsRNA injected crabs; solid bars = control saline crabs. G = granulocytes; SG = semigranulocytes; and, H = hyaline cells. (TIF) [file pone.0136916.s005.TIF]

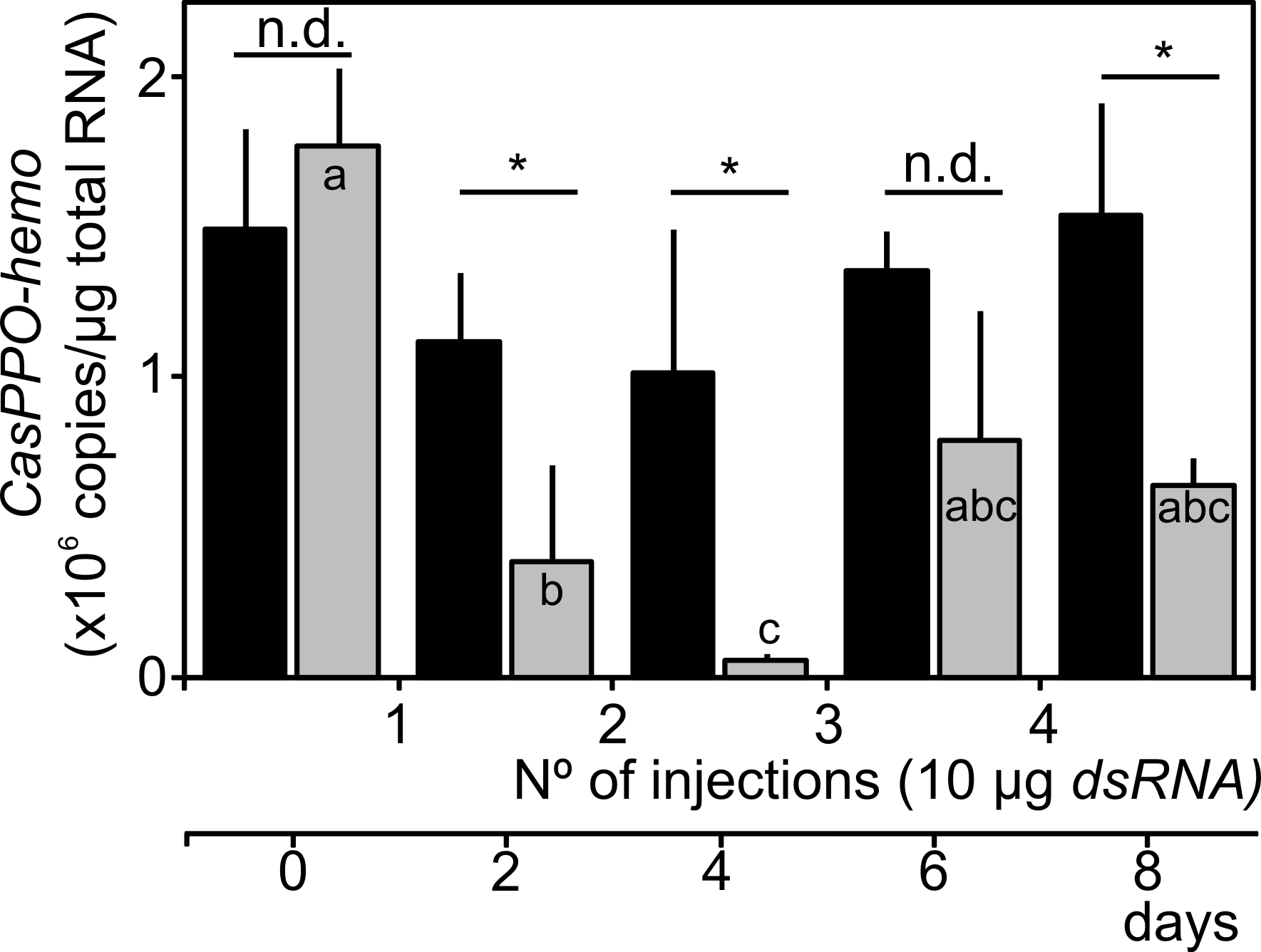

Supplement: S6 Fig — The intermolt crabs received 10 μg of CasPPO-hemo-dsRNA every other day. CasPPO-hemo transcripts are reduced by ~90% after the second injection and ~60% after 4 injections, compared to controls that received crustacean saline. (TIF) [file pone.0136916.s006.TIF]
