# Supplementary material for: The Involvement of Hemocyte Prophenoloxidase in the Shell-Hardening Process of the Blue Crab, Callinectes sapidus
Source: PLoS One. 2015 Sep 22;10(9):e0136916. doi: 10.1371/journal.pone.0136916 (PMC4634603; doi:10.1371/journal.pone.0136916)
Supplement: S1 Table — No significant differences (P > 0.05) are observed using two way ANOVA. (PDF) [file pone.0136916.s007.pdf]

S1 Table. **Hemocyte abundance (cells/ml hemolymph) 24 hrs after ecdysis in *CasPPO-hemo-dsRNA* injected and control crabs**. No significant differences (*P* > 0.05) are observed using two way ANOVA.

Hemocytes	*dsRNA* (n=5)	Control saline (n=4)	Control *dsRNA* (n=5)	Significance
Granulocytes	$8.8 \pm 3.1 \times 10^5$	$6.6 \pm 2.3 \times 10^5$	$8.6 \pm 1.2 \times 10^5$	n.d.
Semigranulocytes	$1.7 \pm 0.6 \times 10^6$	$2.1 \pm 0.5 \times 10^6$	$2.2 \pm 0.6 \times 10^6$	n.d.
Hyaline	$1.4 \pm 0.7 \times 10^6$	$1.8 \pm 0.6 \times 10^6$	$2.0 \pm 0.7 \times 10^6$	n.d.
Total	$5.5 \pm 1.9 \times 10^6$	$5.2 \pm 1.7 \times 10^6$	$5.1 \pm 1.5 \times 10^6$	n.d.
