# Supplementary material for: The Involvement of Hemocyte Prophenoloxidase in the Shell-Hardening Process of the Blue Crab, Callinectes sapidus
Source: PLoS One. 2015 Sep 22;10(9):e0136916. doi: 10.1371/journal.pone.0136916 (PMC4634603; doi:10.1371/journal.pone.0136916)
Supplement: S1 Protocol — (PDF) [file pone.0136916.s008.pdf]

HLS was prepared with the hemocytes of juvenile crabs at intermolt stage, as described [1] and further precipitated using saturated ammonium sulfate (SAS) [2–5]: SAS was added to HLS at 30% final solution (v/v) and mixed on ice for 20 min by constant stirring (~1000 rpm), centrifuged at 15,000 g at 4ºC for 20 min and then, the protein pellet was retrieved and resuspended in 50 or 100 µl of ice-cold working buffer. The remaining solution was used as source for a new ammonium sulfate precipitation at 50, and 65%. The protein concentration of each SAS fraction was measured and evaluated in its PO activity as described [1].

HLSs (2 µg) were separated on a 10% SDS-PAGE. The proteins were transferred onto a nitrocellulose membrane (Bio-Rad) and further processed using the procedure as described [6]. In brief, the membrane was blocked with 7% nonfat milk (NFM) in PBST (1X PBS, 0.05% Tween 20) at RT for 2 h. The primary antibody (α-CasPPO-hemo; 1: 2,000 dilutions in NFM+PBST) was incubated overnight at 4ºC. After washing with PBST (three times per 20 min each), the membrane was then incubated with a secondary antibody, horseradish peroxidase (HRP) conjugated goat anti-rabbit IgG (Jackson Immuno-Research) at 1:10,000 dilutions for 1 hr at RT. The signals on the autoradiography film (HyBlot CL, Denville Scientific Inc) were detected by chemiluminescence using luminol as a substrate.

References

1. Alvarez J V, Chung JS. Cloning of prophenoloxidase from hemocytes of the blue crab, *Callinectes sapidus* and its expression and enzyme activity during the molt cycle. Fish Shellfish Immunol. 2013;35: 1349–1358.

2. Chase MR, Raina K, Bruno J, Sugumaran M. Purification, characterization and molecular cloning of prophenoloxidases from *Sarcophaga bullata*. Insect Biochem Mol Biol. 2000;30: 953–967.

3. Ashida M, Söderhäll K. The prophenoloxidase activating system in crayfish. Comp Biochem Physiol Part B Comp Biochem. 1984;77: 21–26.

4. Ashida M, Kinoshita K, Brey PT. Studies on prophenoloxidase activation in the mosquito *Aedes aegypti* L. Eur J Biochem - FEBS. 1990;188: 507–15.

5. Martin E. Purification of enzyme phenoloxidase from freshwater crab, *Barytelphusa cunicularis* and its kinetic study. World J Zool. 2011;6: 357–359.

6. Ai H-S, Liao J-X, Huang X-D, Yin Z-X, Weng S-P, Zhao Z-Y, et al. A novel prophenoloxidase 2 exists in shrimp hemocytes. Dev Comp Immunol. 2009;33: 59–68.
